# Poor Motor Performance – Do Peers Matter? Examining the Role of Peer Relations in the Context of the Environmental Stress Hypothesis

**DOI:** 10.3389/fpsyg.2020.00498

**Published:** 2020-04-07

**Authors:** Olivia Gasser-Haas, Fabio Sticca, Corina Wustmann Seiler

**Affiliations:** ^1^Marie Meierhofer Children’s Institute, Associated Institute of the University of Zurich, Zurich, Switzerland; ^2^Department of Pre-Primary and Lower Primary Level, Zurich University of Teacher Education, Zurich, Switzerland

**Keywords:** motor performance in daily activities, internalizing problems, peer problems, popularity, friendship quality

## Abstract

The aim of the current study was to investigate pathways of the Environmental Stress Hypothesis concerning the role of peer relations in the context of poor motor skills. First, we examined (1) the mediating role of peer problems in the association between motor performance in daily activities and internalizing problems as a main pathway of the Environmental Stress Hypothesis. Furthermore, we explored the role of (2) children’s popularity as a mediator and (3) best friendship quality as a moderator path of the effect of motor performance on both peer problems and internalizing problems. The non-clinical sample of the present study consisted of 189 children (48.6% females) aged 9–11 years (M_age_ = 9.69, SD_age_ = 0.46). Parents reported on their child’s motor performance in daily activities by completing the Developmental Coordination Disorder Questionnaire. The Strengths and Difficulties Questionnaire was used to assess peer problems as well as internalizing problems. The Self Description Questionnaire provided a measure of children’s self-reported popularity. The Friendship Quality Questionnaire was used to investigate children’s best friendship quality. Results of a structural equation model suggest that peer problems fully mediated the association between the motor performance in daily activities and both popularity and internalizing problems. However, no evidence for the mediating effect of popularity in the association between peer problems and internalizing problems was found. Further, best friendship quality had a non-significant moderating effect on the relation between peer problems and internalizing problems. The mediating role of peer problems highlights the importance of peer relations in the motor performance of daily activities. Schools and psychomotor interventions were suggested as practical implications to support children with poor motor performance in their relationship with their peers and to improve their motor performance in daily activities.

## Introduction

About 5–6% of all school children have motor impairments without any pathophysiology (Schoemaker et al., 2006; Lingam et al., 2012). Affected children have trouble carrying out everyday motor activities such as getting dressed, playing ball, or riding a bicycle and generally tend to be slow, inaccurate, and clumsy in daily motor activities. Two concepts exist that describe children with motor impairments: The clinical concept of Developmental Coordination Disorder (DCD) and the less restrictive concept of “poor motor skills”. The distinction between these two concepts will be outlined in the following and the focus of one of these two concepts for the present study will be explained.

DCD is a developmental disorder characterized by impaired motor coordination that has an undesirable impact in both the academic field and everyday life (American Psychiatric Association, 2013). Neurological disorders such as cerebral palsy must be ruled out (American Psychiatric Association, 2013). In the last 20 years, knowledge about DCD in children has greatly increased (Schoemaker et al., 2006). However, DCD has remained an unknown and underrecognized disorder (Kennedy-Behr et al., 2013). Furthermore, there is no effective instrument for detecting all criteria that are required for a DCD diagnosis, which is why the term DCD is often used incorrectly and inconsistently. As a means of establishing a less clinical concept, Mancini et al. (2019) introduced the term “poor motor skills”. Poor motor skills are not to be understood as a diagnosis, but rather as an umbrella term that encapsulates children who show poorer motor skills than typically developing peers. Independently of whether the motor skills were identified by a full DCD diagnose or focus mainly on motor performance in daily activities. For the present study, the use of this less restrictive term is appropriate because of (a) the inclusion of studies that have or have not used the term DCD in a diagnostic sense, (b) the inclusion of children who are yet to be correctly identified, and (c) the inclusion of children who are just outside the range of a DCD but still struggle with the negative consequences of poor motor skills.

While poor motor skills are not a stressor in the classical sense, the permanent inability to control coordinated motor behaviors leads to a visible dysfunction in the performance in daily activities and this could reasonably be termed as a source of stress (Cairney et al., 2013). A range of far-reaching social and emotional consequences are the result of this stress and endanger the development of psychosocial well-being (Skinner and Piek, 2001; Mancini et al., 2016).

Children with poor motor skills often experience negative and disparaging reactions of their peers. Peers often denigrate, isolate, and stigmatize affected children because of their visible dysfunction and tease, ridicule and bully them (Losse et al., 2008; Cairney et al., 2013; McIntyre et al., 2015; Tal Saban and Kirby, 2019). This might partly explain why children with poor motor skills tend to have fewer playmates, to be ignored by peers more often, and to spend more time alone or watching others as they play (Schoemaker and Kalverboer, 1994; Smyth and Anderson, 2000; Livesey et al., 2011; Cairney et al., 2013). For example, Smyth and Anderson (2000) showed that children with DCD aged between 6 and 10 years spent more time alone and were more often onlookers, than children without DCD. Children’s social involvement is thus affected and interfered, which might result in a longstanding reduced quality of life (Rigoli et al., 2017; Zwicker et al., 2018; Mancini et al., 2019).

As emotional consequences, children with poor motor skills often struggle with the direct consequences in daily activities and with their own negative feelings about it. They report lower self-esteem and self-efficacy (Losse et al., 2008; Cairney et al., 2013; McIntyre et al., 2015; Tal Saban and Kirby, 2019) and more internalizing problems (i.e., anxious and depressive symptoms) (Cairney et al., 2013; Wagner et al., 2016; Mancini et al., 2019). Internalizing problems are particularly frequent mental health problems among children with poor motor skills (Cairney et al., 2013; Wagner et al., 2016; Mancini et al., 2019). Schoemaker and Kalverboer (1994) concluded that clumsy children aged between 6 and 9 years are more introverted than children without clumsiness. Wagner et al. (2016) found in their longitudinal study that primary school children with gross motor coordination problems have a 1.73 times higher risk of emotional problems in adolescence than their classmates without gross motor coordination problems. Mancini et al. (2019) showed in their integrative research review that overall, the effect sizes between motor skills and internalizing problems in community samples are small to medium.

**FIGURE 1:**
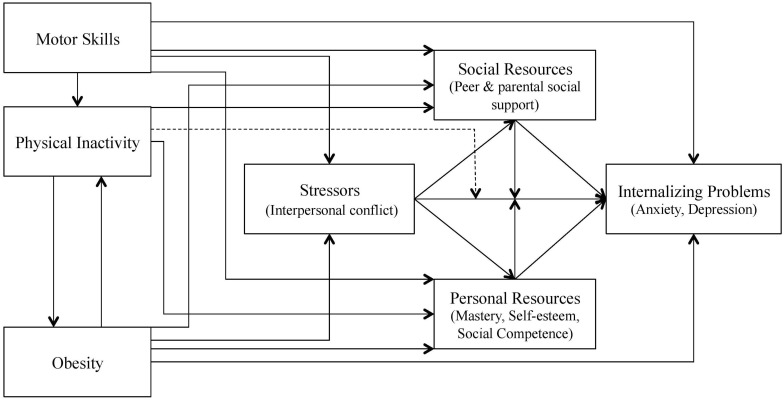
The adapted Environmental Stress Hypothesis by Mancini et al. (2016).

To understand the complex interaction of poor motor skills and internalizing problems, Cairney et al. (2010, 2013) developed the theoretical framework model named Environmental Stress Hypothesis for a DCD-population. Mancini et al. (2016) adapted the Environmental Stress Hypothesis by modifying it for general (poor) motor skills, detached from any DCD diagnosis (see Figure 1). The core assumption of the Environmental Stress Hypothesis is that poor motor skills (i.e., primary stressor) increase the probability of interpersonal conflicts (i.e., secondary stressors) and ultimately lead to an increased risk for the occurrence of internalizing problems (Wagner et al., 2016). Social and personal resources act primarily as mediators or moderators in the association between secondary stressors and internalizing problems. Thus, the association between poor motor skills and internalizing problems is assumed to be largely indirect. Psychosocial environmental factors, such as peer problems, are conceptualized as mediators, which aligns with studies showing that they are a main predictor of psychological disorders such as depression or anxiety (Boivin et al., 1994, 1995; Burks et al., 1995; Cairney et al., 2013). Empirical evidence of this indirect association is growing. For example, Mancini et al. (2016, 2018a, 2018b) and Wagner et al. (2012, 2016) found that peer problems mediated the association between motor skills and internalizing problems. Several studies (e.g., Pearsall-Jones et al., 2011) pointed out that the influence of environmental factors, such as negative peer interactions, play an important mediating role in the relationship between poor motor skills and internalizing problems (Piek et al., 2007, 2010; Cairney et al., 2010, 2013). In sum, unique experienced environmental factors of the children with poor motor skills appear to be more responsible for internalizing problems than the poor motor coordination itself (Mancini et al., 2016). However, these experienced environmental factors of children with poor motor skills are still insufficiently researched.

Forming relationships with peers is a major developmental task, especially in middle childhood (Havighurst, 1976). Children have an interest to be accepted, to have friends, and to be popular. Popular students are often well-liked and perform well in many different activities, in sports and games (Cillessen and Berg, 2012). Children with peer problems have often fewer playmates and are more likely to be bullied and teased by peers (Goodman, 1997). As a result, children with peer problems are often less popular among their peers. According to Prinstein et al. (2018), the more the children are disliked and unpopular, the more internalizing problems they have. The popularity of children can be assessed by others, for example by classmates in social network analyses, or by the child itself, in the sense of self-perceived popularity. Studies have shown that self-perceived likeability and popularity is a much stronger predictor of depression than for example current peer rejection, assessed by other children (Panak and Garber, 1992; Prinstein et al., 2018). The mediating role of popularity has not yet been examined in the context of poor motor skills, peer problems, and internalizing problems. This represents a desideratum for research within the framework of the Environmental Stress Hypothesis.

In the age between nine and eleven, the focus with peers lies on the need for friendships as characterized by mutual understanding, closeness, and recognition (Traub, 2006). According to Sullivan (1953), close friends are of great relevance to child development because they help to question the view of the world as well as of oneself and to build a realistic self-image. There is evidence that children who are popular and well-accepted are more likely to find a good best friend because their “pool” of potential friends is simply greater than that of non-popular and rejected children (Bukowski and Hoza, 1989; Bukowski et al., 1996; Nangle et al., 2003; Schneider, 2016). Moreover, popular children, as well as their potential friends, often have better social skills and can take advantage of these opportunities to build lasting, high-quality relationships (Parker and Asher, 1993; Asher et al., 1996; Nangle et al., 2003; Cairney et al., 2013). But that’s just one side of what’s being replicated, because best friendship quality can have a protective mechanism even if the child is suffering from peer rejection, unpopularity, or peer victimization (Cuadros and Berger, 2016). Therefore, we examined the moderating role of friendship quality as a social resource. Indeed, friendship quality to the best friend, which is characterized by positive dimensions such as closeness, support, and affection, can buffer negative effects of peer interaction, like peer rejection or victimization, on psychosocial outcomes (Sanderson and Siegal, 1995; Oldenburg and Kems, 1997; Bagwell et al., 1998; Schmidt and Bagwell, 2007; Bagwell and Schmidt, 2011; Cuadros and Berger, 2016; Schneider, 2016; Bukowski et al., 2018). For example, a Swedish study with rejected students showed that those with close friendships reported greater satisfaction in life and in their interpersonal relationships than rejected students without a close friend (Schneider, 2016). Parker and Asher (1993) found significant differences in the reports of rejected children about their loneliness. The variability about the perceived loneliness results from the fact that some rejected children have at least one friend who is an important source of emotional support (Parker and Asher, 1993; Nangle et al., 2003). Having a high-quality best friend has two protective functions against victimization and bullying, a direct and an indirect one (Bagwell and Bukowski, 2018). A high-quality best friend can directly be supportive by defending and intervening in challenging peer situations as well as by offering advice on how to react in those stressful peer situations. Through the emotional support and the joint coping of peer victimization experiences, friends can promote resilience and might weaken the negative link between peer victimization and mental health outcomes, like internalizing problems (Bagwell and Bukowski, 2018). A high-quality best friend can indirectly be protective against victimization with his physical presence in many situations. The physical presence leads to fewer opportunities to be bullied and teased because a supportive friend is by the side (Bagwell and Bukowski, 2018). Furthermore, having a high-quality best friend helps to develop own social skills and competencies like emotion regulation skills, and thus foster a child’s positive social reputation (Bagwell and Schmidt, 2011; Bagwell and Bukowski, 2018). To this effect, a high-quality best friend can indirectly protect against victimization by minimizing the risk (Bagwell and Bukowski, 2018). Little is known about the protective effect of best friendship quality within the Environmental Stress Hypothesis framework.

The Environmental Stress Hypothesis framework consists of many direct and indirect, main and conditional paths. Therefore, studies often evaluate a subset of pathways embedded in the superordinate framework model (Mancini et al., 2019). Peer-related stressors and resources, such as victimization, social support, and empathy were already tested within this framework, but in contrast to the present study, these studies have often examined direct effects rather than indirect and conditional effects that are fundamental to the Environmental Stress Hypothesis (Mancini et al., 2016, 2019; Tal Saban and Kirby, 2019). Mancini et al. (2019), for example, concluded that moderator variables are still underexplored and have to be evaluated in future research projects. In addition, popularity and friendship quality are in the context of motor skills, peer problems, and internalizing problems still too little researched.

The present study aimed to investigate peer-related stressor as well as protective factors, like social and personal resources, in a sample of 9–11 years old children. The age between 9 and 11 years is not only crucial for building and maintaining popularity and friendships, but the motor performance in daily activities and their social appraisal starts to increase dramatically at this age, as Leversen et al. (2012) showed in their study.

**FIGURE 2:**
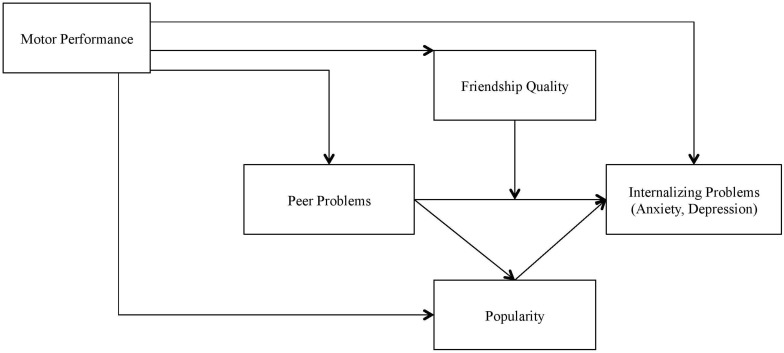
The working model within the framework of the Environmental Stress Hypothesis with the paths tested in the study.

The children have all a non-clinical background. Even without an existing DCD diagnosis, poor motor skills can be sufficiently stressful and lead to internalizing problems and general mental health problems of children. We hypothesized, that (1) the motor performance in daily activities has a negative (i.e., undesirable) effect on internalizing problems in a non-clinical sample, and that this effect is largely mediated by peer problems. Furthermore, we examined, (2) whether children’s popularity, as a personal resource, mediated the relationship between peer problems and internalizing problems, and (3) if best friendship quality, as a social resource, moderated the positive effect between peer problems and internalizing problems. Gender and age were included as covariates. Figure 2 shows the paths tested within the framework model of the Environmental Stress Hypothesis. The motor performance in daily activities is modeled as the independent and internalizing problems as the dependent variable. Peer problems are modeled as a mediator between the motor performance in daily activities and internalizing problems and popularity as a mediator between peer problems and internalizing problems. Best friendship quality is included in the model as a moderator in the association between peer problems and internalizing problems. Covariates are not displayed in the working model.

## Materials and Methods

### Participants

The present study was part of the project “Long-term effects of early family risk on children’s maladjustment and self-efficacy: individual, familial and extra-familial protective processes” (2016–2019) that belongs to the project “Promoting early learning and resilience through a strengthening learning dialogue – A project for promotion and professionalization of early childhood education in Swiss childcare centers” (2009–2012) (Wustmann Seiler et al., 2017). The study encompasses three measurement waves, from early childhood to middle school age. In 2009, 293 children (47.9% female) and their parents from 25 day care centers in Switzerland were recruited and interviewed. At the first measurement wave, the children were aged 2–4 years (T1; M_age_ = 2.81, SD_age_ = 0.55). One year later, in 2010, the same children (T2; M_age_ = 3.76; SD_age_ = 0.49; 47.3% female) at the age of 3–5 years and their parents were enrolled to participate in the second measurement wave. Six years later, in 2016, 189 children (48.6% female) aged 9–11 years (T3; M_age_ = 9.69, SD_age_ = 0.48) and their parents took part in the study. The current study only refers to the third and last measurement wave. While the first two measurement waves concentrated on familial risk factors, socio-emotional and behavioral strengths and difficulties, only the third and final measurement wave focused on poor motor skills and peer relation factors in relation to internalizing problems. This developmental time window is of central importance because middle childhood is when children with poor motor skills begin to stand out. As a consequence, their peers might begin to react negatively, as Schoemaker and Kalverboer (1994) showed in their study with 6–7 years old children. Thus, the social comparison of motor skills in the school setting leads to social pressure, which might in turn be linked to internalizing problems. For this reason, friendship quality and poor motor skills were introduced at the third measurement wave of the present study. At T3 the participating families were mainly from the upper middle class: 70% of the mothers and 74% of the fathers had a university degree. 12% of the children had a foreign language background.

A total of 103 children and their parents dropped out of the study. The analyses of missing data showed that children who participated in both T2 and T3 had comparable scores of internalizing problems (ß = −0.05; *p* = 0.71) and peer problems (ß = −0.07; *p* = 0.81).

### Measures

#### Motor Performance in Daily Activities

The children’s motor performance in daily activities was assessed by parents using the Developmental Coordination Disorder Questionnaire (DCDQ-G; Kennedy-Behr et al., 2013). The DCDQ-G was developed to contribute to the identification of DCD in children whereby parents compare their child’s coordination in everyday motor activities to that of their children’s peers. The DCDQ-G is the German version of the DCDQ-07 and consists of 15 items that are assigned to three subscales: control during movement (e.g., “Your child throws a ball in a controlled and accurate fashion”), fine motor and handwriting (e.g., “Your child’s printing or writing letters, numbers and words is legible, precise and accurate or, if your child is not yet printing he or she colors and draws in a coordinated way and makes pictures that you can recognize”), and general coordination (e.g., “Your child is quick and competent in tidying up, putting on shoes, tying shoes, dressing, etc.”). The items are rated on a 5-point scale from 1 [not true (for my child)] to 4 [true (for my child)]. For the present analysis, all items of the DCDQ-G (McDonald’s Omega = 0.72–0.87) were used to assess the motor performance in daily activities. Previous studies indicated the DCDQ has good psychometric properties (Wilson et al., 2009; Kennedy-Behr et al., 2013). Wilson et al. (2009), for example, showed that internal consistency was high (Cronbach’s Alpha = 0.94). Furthermore, they provided evidence of construct validity as well as concurrent validity, as DCDQ scores and the scores of the Movement Assessment Battery for Children (MABC; Henderson and Sugden, 1992) correlated with each other (*r* = 0.55). Also, Schoemaker et al. (2006) reported results that gave credit to the notion of the DCDQ as a valid and reliable questionnaire for assessing the daily motor performance of 4–12 years old children.

#### Peer Problems and Internalizing Problems

The Strengths and Difficulties Questionnaire (SDQ; Goodman, 1997) is a widely used questionnaire to identify emotional and behavioral strengths and difficulties of 3–16 year olds. Numerous national and international studies yielded results on the psychometric properties of the SDQ and have shown satisfactory reliability (Goodman, 2001; Koglin et al., 2007; Gómez-Beneyto et al., 2013). For example, in the study of Koglin et al. (2007) the Cronbach’s Alpha was between 0.73 and 0.86 for all subscales.

The SDQ consists of five subscales, each with five items: conduct problems, hyperactivity, peer problems, prosocial behavior, and emotional problems. Emotional problems are often defined as internalizing problems, as in the current study. The parents responded to the SDQ on a 3-point scale ranging from 1 (not true) to 3 (certainly true). This study focused on internalizing problems (e.g., “Many fears, easily scared”) and peer problems (e.g., “Rather solitary, prefers to play alone”). A confirmatory factor analysis was carried out with the scale of internalizing problems and the scale of peer problems. The two items for the latent variable internalizing problems “Often complains about headaches, stomach-aches or sickness” and “Nervous in new situations, easily loses confidence” had loadings of 0.47. Given that this equals an amount of explained variance of less than 25%, these items were removed from the scale of internalizing problems, which is in line with Hulland (1999) and Hair (2014). Regarding the latent variable peer problems, the item “Has at least one good friend” was removed from the analysis, because of the low loading of 0.41. As a result, the McDonalds’ Omega reliability was 0.67 for internalizing problems and 0.59 for peer problems. Given the just acceptable reliability, internalizing and peer problems were kept in the model as latent variables.

#### Popularity

The Self Description Questionnaire is one of the most commonly used instruments for measuring the multiple dimensions of children’s self-concept, with a Cronbach’s Alpha internal consistency values between 0.80 and 0.92 (SDQ-I; Marsh, 1990; Arens et al., 2011). Children completed the subscale self-concept peer relations. Three items of this subscale measured children’s self-perceived popularity on a 5-point Likert scale. Children were asked if a set of sentences tapping into popularity were true (4), mostly true (3), sometimes true (2), mostly false (1), or false (0). The following three items were used: “I am popular among other kids of my age,” “Other children would like to have me as a friend,” and “Most other kids like me.” McDonalds’ Omega for popularity was 0.85.

#### Friendship Quality

The best friendship quality was assessed using the well-established Friendship Quality Questionnaire (FQQ; Parker and Asher, 1993). The scale consists of 40 items with six subscales, five positive components (Help and Guidance, Intimate Exchange, Validation and Caring, Companionship and Recreation, and Conflict Resolution) and one negative component (Conflict and Betrayal). Parker and Asher (1993) estimated a Cronbach’s Alpha internal consistency value between 0.73 and 0.90 for the various subscales. Previously, the children were asked to write down the name of their best friend to make a clear reference. In the present study, the children answered the items of four positive subscales to assess their self-perceived *positive* friendship quality concerning their best friend: Help and Guidance (e.g., “Do special favors for each other”), Intimate Exchange (e.g., “Talk about the things that make us sad”), Validation and Caring (e.g., “Makes me feel good about my ideas”), and Conflict Resolution (e.g., “Make up easily when we have a fight”) (McDonald’s Omega = 0.74–0.88). The answer format consisted of a 5-point Likert scale from 1 “Not at all true” to 4 “Absolutely true.”

### Procedure

The children aged 9–11 years received age-appropriate written information about the objectives and procedures of the study. Also, their parents received written information and in addition verbal information. Both, the children and their parents were informed about their right to quit their participation at any time without giving reasons. The participating children and their parents had to give their written informed consent, agreeing to cooperate and giving permission for an interview and to administer a questionnaire. A trained research assistant carried out the interviews and questionnaires with the families at their houses. The research assistant interviewed the parents first and the child afterward. The parents answered more sensitive questions in a written questionnaire. The children and their parents were informed, that after completing data collection, the data were anonymized and used for research purposes only.

All procedures performed in the study were in accordance with the ethical standards of the Swiss legislation for research with human participants and in accordance with the declaration of Helsinki 1964 and its subsequent amendments. Neither at the time of the original study nor at the time of the follow-up study ethical approval was required as the local ethics committee of the Swiss Ethics Committees on research involving humans declared.

### Analysis Strategy

In order to analyze the full structural equating model (SEM) with the moderating role of best friendship quality and the mediating role of peer problems and popularity in the Environmental Stress Hypothesis framework we analyzed all data using Mplus Version 8.0 (Muthén and Muthén, 1998-2017). All constructs in the model were modeled as latent variables, except best friendship quality. Best friendship quality was modeled manifestly to reduce complexity. The full information maximum likelihood (FIML) for missing data was applied. The data of this article can be found in the Supplementary Material of this article.

The analyses of the current study were divided into three steps, all realized in one structural equation model. In a first step, the main effect of the motor performance in daily activities on internalizing problems was tested and peer problems were specified as a mediator variable between the motor performance in daily activities and internalizing problems. The latent variable for internalizing problems was modeled with the retained three items as manifest indicators. The three mean scores of each subscale of the DCDQ were built with all corresponding items and modeled as indicators of the latent variable motor performance in daily activities. Motor performance in daily activities was then introduced as a predictor of internalizing problems. Peer problems were modeled as a latent variable with the four retained items as manifest indicators and added to the model as a further predictor of internalizing problems. An indirect path from motor performance in daily activities to peer problems and to internalizing problems was added to the model. In a second step, popularity, consisting of three items, was added as a mediator variable between peer problems and internalizing problems. In a third step, best friendship quality was included as a manifest moderator of the relation between peer problems and internalizing problems. To test for the moderation effect of best friendship quality, the latent orthogonalization method was applied (Little, 2013). Finally, children’s gender and age were included as manifest predictors of internalizing problems, peer problems, and popularity and were allowed to correlate with the motor performance in daily activities, best friendship quality, and its interaction. All latent variables were modeled with the effect coding method (Little et al., 2006). The over-identified model fitted the data very well [χ^2^(147) = 160.52; CFI = 0.97; RMSEA = 0.02; SRMR = 0.05].

## Results

**FIGURE 3:**
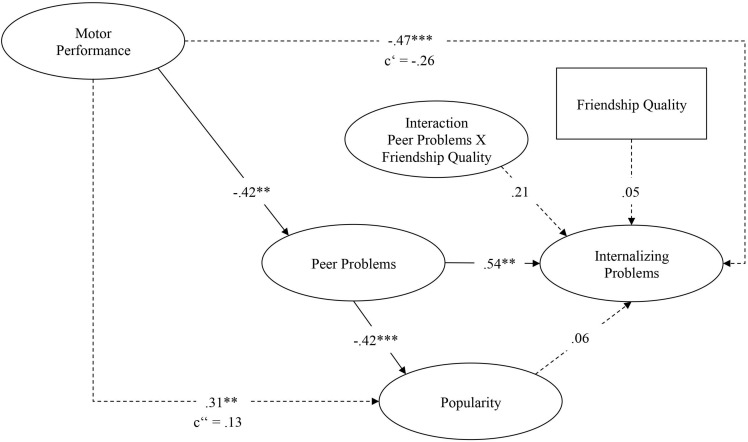
Standardized results of the structural equation model. Model fit (χ^2^ = 160.52; *df* = 147; CFI = 0.97; RMSEA = 0.02; SRMR = 0.05). *p < 0.05; **p < 0.01; ***p < 0.001.

Table 1 shows intercorrelations between all study variables as well as their means and standard deviations. The results of the model are reported in Figure 3.

**TABLE 1:** Means, standard deviations, ranges, and intercorrelations between main variables (*N* = 189).

	**M**	**SD**	**Min**	**Max**	**1**	**2**	**3**	**4**	**5**	**6**
1 Motor performance	3.34	0.44	1.80	4.00	1					
2 Peer problems	1.19	0.20	1.00	2.00	−0.46***	1				
3 Internalizing problems	1.27	0.29	1.00	2.67	−0.44***	0.59***	1			
4 Popularity	2.84	0.74	0.33	4.00	0.28**	−0.44***	−0.24*	1		
5 Friendship quality	2.91	0.75	0.75	4.00	0.11	−0.14	0.00	0.42***	1	
6 Gender (male)	–	–	–	–	−0.29**	0.25**	0.06	−0.01	−0.23**	1
7 Age (years)	9.69	0.48	8.85	11.05	−0.01	−0.01	0.04	−0.11	−0.10	0.05

*Number 1–4 were latently modeled and number 5–7 manifestly modeled.* *p < 0.05; **p < 0.01; ***p < 0.001.

### The Mediating Role of Peer Problems

The analysis showed that peer problems mediated the association between the motor performance in daily activities and internalizing problems. While the total effect of the motor performance in daily activities on internalizing problems was negative and medium to large (ß = −0.47, *p* < 0.001), the effect with the addition of the mediator peer problems was negative, medium in size and non-significant (ß = −0.26, *p* = 0.13). In other words these results suggest that children with poor motor performance in daily activities had more internalizing problems because they had more peer problems, although the causality of this association cannot be determined here.

### The Mediating Role of Popularity

There was a medium to large effect of peer problems on popularity (ß = −0.42, *p* < 0.001), but an effect very close to zero of popularity on internalizing problems (β = 0.06, *p* = 0.70). Thus, popularity did not mediate the effect of peer problems on internalizing problems. Nevertheless, tests of direct and indirect effects showed a significant full mediating effect of peer problems on the relation between the motor performance in daily activities (β = −0.42, *p* < 0.01) and popularity.

### The Moderating Role of Best Friendship Quality

Best friendship quality had a small to medium non-significant positive moderation effect on the relation between peer problems and internalizing problems (β = 0.21, *p* = 0.15). Moreover, no significant main effect of best friendship quality on internalizing problems was found (β = 0.05, *p* = 0.68). Therefore positive best friendship quality protects just up to a certain point. After that point, it is more of a risk factor.

### Covariates Gender and Age

The results showed that girls were found to have higher scores on friendship quality than boys (β = −0.23, *p* < 0.01) and also higher scores on the motor performance in daily activities than boys (β = −0.29, *p* < 0.01). No association was found between age and other variables.

## Discussion

### Summary

The aim of the present study was to examine the role of peer relations in the Environmental Stress Hypothesis framework. We investigated (1) the mediating role of peer problems in the association between the motor performance in daily activities and internalizing problems in a non-clinical sample as a main pathway of the Environmental Stress Hypothesis. Furthermore, we examined (2) the role of children’s popularity as a mediator of the relationship between peer problems and internalizing problems and (3) the best friendship quality as a moderator of the effect of peer problems on internalizing problems.

In line with Wagner et al. (2016, 2012) and Mancini et al. (2016), we were able to show that the effect of motor performance on internalizing problems was indirect rather than direct: Poor motor performance in daily activities is linked to higher internalizing problems, which might be partly explained by peer problems, although the causality of this association cannot be determined at this point. In accordance with Rigoli et al. (2017), our results indicated that even without an existing DCD diagnosis, poor motor performance in daily activities can be very stressful and lead to internalizing problems.

In contrast to results reported by Panak and Garber (1992), we found no support for the mediating role of popularity in the association between peer problems and internalizing problems. Results showed that children with peer problems perceived themselves as less popular. But less popular children did not show more internalizing problems than their more popular peers. Although, in line with other studies, the intercorrelation between popularity and internalizing problems was medium and significant (Bell-Dolan et al., 1995), popularity had no predictive value over and above peer problems.

Popularity of children is linked to their motor performance. We found that poor motor performance enhances peer problems and these in turn influence child’s unpopularity. To the best of our knowledge, this study is the first to show that children with poor motor performance are more likely to perceive themselves as being unpopular in response to peer problems. Children with peer problems are more frequently bullied and teased by peers and usually play more time alone (Goodman, 1997).

Best friendship quality had a small but non-significant positive moderation effect on the relation between peer problems and internalizing problems. As no main effect of best friendship quality on internalizing problems was found, the small buffering effect of best friendship quality on internalizing problems might only be protective up to a certain point. From that point, having a best friendship quality is more a risk factor, than a protective factor, and children who have peer problems have an increased risk having internalizing problems, if they have a best friendship quality. A possible explanation can be found in the best friend attributes. Having a high-quality best friend who is itself high on peer problems may exacerbate the association between peer problems on internalizing problems (Bagwell and Bukowski, 2018). Instead of reducing the internalizing problems, an increase in the problems occurs. For the buffering function of best friendship quality having a well-adjusted friend seems to be necessary (Bagwell and Bukowski, 2018). For this reason, more information about the friend’s characteristics is required.

### Strengths and Limitations

This is the first study that examines both, mediated and moderated effects in the Environmental Stress Hypothesis framework. The major strength of the contribution lies in the complex modeling of the interplay among the various constructs along the lines of the theory. Furthermore, the addition of peer problems, popularity, and best friendship quality in the model, as important peer factors in middle childhood, are other strengths of the contribution. The study showed support for the Environmental Stress Hypothesis framework and contribute to the current state of research on motor performance in connection with internalizing problems and peer relations. Even if not all investigated pathways were significant, the pattern of the model was clearly visible and the effect sizes were meaningful.

There are also some limitations that need to be considered. First, only cross-sectional associations could be reported. Thus, no cause-and-effect relationships could be identified over time. Furthermore, according to usual guidelines (Muthén and Muthén, 2002), the statistical power in the study is limited. One reason for this is the limiting sample size for the model. Accordingly, the interpretation of the results in this study should be treated with caution. Studies with a longitudinal design and a large representative sample size are required to replicate the results. In addition, there is no high-risk DCD group. None of the children had a DCD to their own knowledge. Furthermore, the DCDQ is a self-report of the parents. Although studies have highlighted the correlation between the DCDQ and standardized motor assessments, there is some evidence that the values of the two measures differ, as a questionnaire never can replace a standardized motor assessment (Schoemaker et al., 2006). Additionally, we could not test whether or not the best friendship was based on reciprocity nor whether the best friend is a real or an imaginary companion (e.g., Taylor et al., 2010; Gleason, 2017), as children assessed their friendship quality by themselves. However, the subjective assessment of the children’s friendship quality might well be more important than objective friendship quality in the sense of reciprocity, which is already well-established in the field of perceived social support (Uchino, 2009; Eagle et al., 2019). And finally, the child best friend’s characteristics are missing. Children with same motor performance level in daily activities tend to befriend (Peters et al., 2010).

### Implications for Practice

The findings of this study lead to some suggestions for children with poor motor performance to protect them from the far-reaching consequences of their poor motor performance. In these contexts, peer relations play a key role to prevent and reduce negative impacts of motor performance. Schools and psychomotor interventions are decisive for this support.

#### Implications for School

The influence of motor performance on multiple factors, such as the level of self-perception or peer support, in children aged 9–11 years, is considerable. Two implications for school should be mentioned. First, teachers need to have knowledge of the consequences and complexities of children with poor motor performance and children affected by DCD, which are until now frequently undiagnosed (Cairney, 2015). A lack of understanding can lead to children being misunderstood as lazy, or defiant (Fox and Lent, 1996; Cairney et al., 2013; Cairney, 2015). Second, peer problems often arise and manifest themselves in the school context. Therefore, schools have a responsibility to reduce multiple forms of peer problems. There are several evidence-based programs to prevent and tackle peer problems, such as bullying at school (see for a systematic review of effective school-based prevention programs; Gaffney et al., 2019).

#### Psychomotor Intervention

While schools can address the problems of peers at class and group level, at the individual level therapeutic interventions are needed to support children with poor motor skills in their relationships with peers. According to Cantell et al. (2003), Cairney et al. (2013), and Schoemaker and Wilson (2015), the effects of psychomotor interventions (motor-based in combination with psychological interventions) for children with poor motors skills are undisputed. On the one hand, children can experience success in movement and action, combined with positive emotions and joy in psychomotor therapies. On the other hand, they receive opportunities to learn the interaction with their peers and build positive relationships. Although psychomotor interventions and therapies cannot compensate for all adaptive, qualitative differences in performance, offering efficient strategies and positive movement experiences helps to prevent the impact of negative experiences to the academic, social, and emotional well-being of affected children (Cantell et al., 2003).

## Conclusion

The present study provides a relevant contribution to the maintenance and extension of the Environmental Stress Hypothesis framework concerning the role of peer relations:

1. The study showed that poor motor performance in daily activities, independent of a given DCD diagnosis, is associated with more peer problems. These peer problems, in turn, are positively linked to a child’s internalizing problems.
2. The present results suggest that children with poor motor performance in daily activities tend to be less popular, indirectly affected through peer problems.
3. The study’s results support the notion that best friendship quality may protect children with peer problems from internalizing problems up to a certain point, although this has to be replicated by future longitudinal studies.

Future research within the Environmental Stress Hypothesis framework is required, especially with moderating effects, to determine the buffering effect of peer problems on internalizing problems for children with poor motor skills.

## Data Availability Statement

The dataset generated for this study is included in the Supplementary Material.

## Ethics Statement

Ethical review and approval was not required for the study on human participants in accordance with the local legislation and institutional requirements. Written informed consent to participate in this study was provided by the participants’ legal guardian/next of kin.

## Author Contributions

CW designed and managed the study, developed the instruments, and recruited the sample. OG-H collected the data, analyzed and interpreted the model, and wrote the first draft of the manuscript. FS was responsible for the entire project data management and interpreted the model in collaboration with OG-H. OG-H, FS, and CW have critically reviewed and revised the manuscript. All authors agreed to release the final version.

## Conflict of Interest

The authors declare that the research was conducted in the absence of any commercial or financial relationships that could be construed as a potential conflict of interest.

## References

[B1] American Psychiatric Association (2013). *Diagnostic and Statistical Manual of Mental Disorders*, 5th edn Philadelphia: American Psychiatric Publishing.

[B2] Arens A. K. Trautwein U. Hasselhorn M. (2011). Erfassung des Selbstkonzepts im mittleren Kindesalter: Validierung einer deutschen Version des SDQ. *Z. Pädagog. Psychol.* 25 131–144. 10.1024/1010-0652/a000030

[B3] Asher S. R. Parker J. Walker D. L. (1996). “Distinguishing friendship from acceptance: implications for intervention and assessment,” in *The Company they Keep. Friendship in Childhood and Adolescence*, eds Bukowski W. M. Newcomb A. F. Hartup W. W. (Cambridge, MA: Cambridge University Press), 366–405.

[B4] Bagwell C. L. Bukowski W. M. (2018). “Friendship in childhood and adolescence: features, effects, and processes,” in *Handbook of Peer Interactions, Relationships, and Groups*, 2nd Edn, eds Bukowski W. M. Laursen B. Rubin K. H. (New York, NY: The Guilford Press), 371–390.

[B5] Bagwell C. L. Newcomb A. F. Bukowski W. M. (1998). Preadolescent friendship and peer rejection as predictors of adult adjustment. *Child Dev.* 69 140–153. 10.1111/j.1467-8624.1998.tb06139.x 9499563

[B6] Bagwell C. L. Schmidt M. E. (2011). *Friendships in Childhood and Adolescence.* New York, NY: Guilford Press.

[B7] Bell-Dolan D. J. Foster S. L. Christopher J. S. (1995). Girls’ peer relations and internalizing problems: are socially neglected, rejected, and withdrawn girls at risk? *J. Clin. Child Psychol.* 24 463–473. 10.1207/s15374424jccp2404_10

[B8] Boivin M. Hymel S. Bukowski W. M. (1995). The roles of social withdrawal, peer rejection, and victimization by peers in predicting loneliness and depressed mood in childhood. *Dev. Psychopathol.* 7 765–785. 10.1017/S0954579400006830

[B9] Boivin M. Poulin F. Vitaro F. (1994). Depressed mood and peer rejection in childhood. *Dev. Psychopathol.* 6 483–498. 10.1017/S0954579400006064

[B10] Bukowski W. M. Hoza B. (1989). “Popularity and friendship: issues in theory, measurement, and outcome,” in *Peer Relationships in Child Development*, eds Berndt T. J. Ladd G. W. (Hoboken, NJ: John Wiley & Sons), 15–45.

[B11] Bukowski W. M. Laursen B. P. Rubin K. H. (eds) (2018). *Handbook of Peer Interactions, Relationships, and Groups*, 2nd Edn New York, NY: The Guilford Press.

[B12] Bukowski W. M. Newcomb A. F. Hartup W. W. (eds) (1996). *The Company they Keep: Friendship in Childhood and Adolescence.* Cambridge, MA: Cambridge University Press.

[B13] Burks V. S. Dodge K. A. Price J. M. (1995). Models of internalizing outcomes of early rejection. *Dev. Psychopathol.* 7 683–695. 10.1017/S0954579400006787

[B14] Cairney J. (ed.) (2015). *Developmental Coordination Disorder, and its Consequences.* Toronto: Universit of Toronto Press.

[B15] Cairney J. Rigoli D. Piek J. (2013). Developmental coordination disorder and internalizing problems in children: the environmental stress hypothesis elaborated. *Dev. Rev.* 33 224–238. 10.1016/j.dr.2013.07.002 26941690

[B16] Cairney J. Veldhuizen S. Szatmari P. (2010). Motor coordination and emotional-behavioral problems in children. [Review] [51 refs]. *Curr. Opin. Psychiatry* 23 324–329. 10.1097/YCO.0b013e32833aa0aa 20520549

[B17] Cantell M. H. Smyth M. M. Ahonen T. P. (2003). Two distinct pathways for developmental coordination disorder: persistence and resolution. *Hum. Mov. Sci.* 22 413–431. 10.1016/j.humov.2003.09.002 14624826

[B18] Cillessen A. H. N. Berg Y. H. M. V. D. (2012). “Popularity and school adjustment,” in *Peer Relationships and Adjustment at School*, eds Ryan A. M. Ladd G. W. (Charlotte: IAP Information Age Publishing), 135–164.

[B19] Cuadros O. Berger C. (2016). The protective role of friendship quality on the wellbeing of adolescents victimized by peers. *J. Youth Adolesc.* 45 1877–1888. 10.1007/s10964-016-0504-4 27230120

[B20] Eagle D. E. Hybels C. F. Proeschold-Bell R. J. (2019). Perceived social support, received social support, and depression among clergy. *J. Soc. Pers. Relationsh.* 36 2055–2073. 10.1177/0265407518776134

[B21] Fox A. M. Lent B. (1996). Clumsy children. Primer on developmental coordination disorder. *Can. Fam. Phys.* 42 1965–1971. PMC2146951 8894243

[B22] Gaffney H. Farrington D. P. Ttofi M. M. (2019). Examining the effectiveness of school-bullying intervention programs globally: a meta-analysis. *Int. J. Bull. Prevent.* 1 14–31. 10.1007/s42380-019-0007-4

[B23] Gleason T. R. (2017). The psychological significance of play with imaginary companions in early childhood. *Learn. Behav.* 45 432–440. 10.3758/s13420-017-0284-z 28707060

[B24] Gómez-Beneyto M. Nolasco A. Moncho J. Pereyra-Zamora P. Tamayo-Fonseca N. Munarriz M. (2013). Psychometric behaviour of the strengths and difficulties questionnaire (SDQ) in the Spanish national health survey 2006. *BMC Psychiatry* 13:95. 10.1186/1471-244X-13-95 23522343 PMC3623874

[B25] Goodman R. (1997). The strengths and difficulties questionnaire: a research note. *J. Child Psychol. Psychiatry* 38 581–586. 10.1111/j.1469-7610.1997.tb01545.x 9255702

[B26] Goodman R. (2001). Psychometric properties of the strengths and difficulties questionnaire. *J. Am. Acad. Child Adolesc. Psychiatry* 40 1337–1345. 10.1097/00004583-200111000-00015 11699809

[B27] Hair J. F. (ed.) (2014). *Multivariate Data Analysis*, 7th Edn London: Pearson.

[B28] Havighurst R. J. (1976). *Developmental Tasks and Education*, 3rd Edn Chicago, IL: McKay.

[B29] Henderson S. E. Sugden D. A. (1992). *Movement Assessment Battery for Children*, 2nd Edn Sidcup: Psychological Corporation.

[B30] Hulland J. (1999). Use of partial least squares (PLS) in strategic management research: a review of four recent studies. *Strateg. Manag. J.* 20 195–204. 10.1002/(sici)1097-0266(199902)20:2<195::aid-smj13>3.0.co;2-7

[B31] Kennedy-Behr A. Wilson B. Rodger S. Mickan S. (2013). Cross-cultural adaptation of the developmental coordination disorder questionnaire 2007 for German-speaking countries: DCDQ-G. *Neuropediatrics* 44 245–251. 10.1055/s-0033-1347936 23716299

[B32] Koglin U. Barquero B. Mayer H. Scheithauer H. Petermann F. (2007). Deutsche Version des Strengths and Difficulties Questionnaire (SDQ-Deu). *Diagnostica* 53 175–183. 10.1026/0012-1924.53.4.175

[B33] Leversen J. Haga M. Sigmundsson H. (2012). From children to adults: motor performance across the life-span. *PLoS One* 7:e38830. 10.1371/journal.pone.0038830 22719958 PMC3377693

[B34] Lingam R. Jongmans M. J. Ellis M. Hunt L. P. Golding J. Emond A. (2012). Mental health difficulties in children with developmental coordination disorder. *Pediatrics* 129 e882–e891. 10.1542/peds.2011-1556 22451706

[B35] Little T. D. (2013). *Longitudinal Structural Equation Modeling.* New York, NY: Guilford Press.

[B36] Little T. D. Slegers D. W. Card N. A. (2006). A non-arbitrary method of identifying and scaling latent variables in SEM and MACS models. *Struct. Equ. Model. Multidiscip. J.* 13 59–72. 10.1207/s15328007sem1301_3

[B37] Livesey D. Lum Mow M. Toshack T. Zheng Y. (2011). The relationship between motor performance and peer relations in 9- to 12-year-old children: motor performance and peer relations. *Child Care Health Dev.* 37 581–588. 10.1111/j.1365-2214.2010.01183.x 21143269

[B38] Losse A. Henderson S. E. Elliman D. Hall D. Knight E. Jongmans M. (2008). Clumsiness in children-do they grow out of it? A 10-year follow-up study. *Dev. Med. Child Neurol.* 33 55–68. 10.1111/j.1469-8749.1991.tb14785.x 1704864

[B39] Mancini V. Rigoli D. Cairney J. Roberts L. D. Piek J. P. (2016). The elaborated environmental stress hypothesis as a framework for understanding the association between motor skills and internalizing problems: a mini-review. *Front. Psychol.* 7:239. 10.3389/fpsyg.2016.00239 26941690 PMC4763061

[B40] Mancini V. Rigoli D. Roberts L. Piek J. (2019). Motor skills and internalizing problems throughout development: an integrative research review and update of the environmental stress hypothesis research. *Res. Dev. Disabil.* 84 96–111. 10.1016/j.ridd.2018.07.003 30054197

[B41] Mancini V. Rigoli D. Roberts L. D. Heritage B. Piek J. P. (2018a). The relationship between motor skills and psychosocial factors in young children: a test of the elaborated environmental stress hypothesis. *Br. J. Educ. Psychol.* 88 363–379. 10.1111/bjep.12187 28884809

[B42] Mancini V. Rigoli D. Roberts L. Heritage B. Piek J. (2018b). The relationship between motor skills, perceived self-competence, peer problems and internalizing problems in a community sample of children. *Infant Child Dev.* 27:e2073. 10.1002/icd.2073 28884809

[B43] Marsh H. W. (1990). *SDQ Manual: Self-Description Questionnaire.* Campbelltown: University of Western Sydney, Macarthur.

[B44] McIntyre F. Chivers P. Larkin D. Rose E. Hands B. (2015). Exercise can improve physical self perceptions in adolescents with low motor competence. *Hum. Mov. Sci.* 42 333–343. 10.1016/j.humov.2014.12.003 25543182

[B45] Muthén L. K. Muthén B. O. (1998). *Mplus User’s Guide*, 8th Edn Los Angeles, CA: Muthén & Muthén.

[B46] Muthén L. K. Muthén B. O. (2002). How to use a monte carlo study to decide on sample size and determine power. *Struct. Equ. Model. Multidiscip. J.* 9 599–620. 10.1207/S15328007SEM0904_8

[B47] Nangle D. W. Erdley C. A. Newman J. E. Mason C. A. Carpenter E. M. (2003). Popularity, friendship quantity, and friendship quality: interactive influences on children’s loneliness and depression. *J. Clin. Child Adolesc. Psychol.* 32 546–555. 10.1207/S15374424JCCP3204_7 14710463

[B48] Oldenburg C. M. Kems K. A. (1997). Associations between peer relationships and depressive symptoms: testing moderator effects of gender and age. *J. Early Adolesc.* 17 319–337. 10.1177/0272431697017003004

[B49] Panak W. F. Garber J. (1992). Role of aggression, rejection, and attributions in the prediction of depression in children. *Dev. Psychopathol.* 4 145–165. 10.1017/S0954579400005617

[B50] Parker J. G. Asher S. R. (1993). Friendship and friendship quality in middle childhood: links with peer group acceptance and feelings of loneliness and social dissatisfaction. *Dev. Psychol.* 29 611–621. 10.1037/0012-1649.29.4.611

[B51] Pearsall-Jones J. G. Piek J. P. Steed L. McDougall M. R. Levy F. (2011). Monozygotic twins concordant and discordant for DCD: two sides to the story. *Twin Res. Hum. Genet.* 14 79–87. 10.1375/twin.14.1.79 21314259

[B52] Peters E. Cillessen A. H. N. Riksen-Walraven J. M. Haselager G. J. T. (2010). Best friends’ preference and popularity: associations with aggression and prosocial behavior. *Int. J. Behav. Dev.* 34 398–405. 10.1177/0165025409343709

[B53] Piek J. P. Barrett N. C. Smith L. M. Rigoli D. Gasson N. (2010). Do motor skills in infancy and early childhood predict anxious and depressive symptomatology at school age? *Hum. Mov. Sci.* 29 777–786. 10.1016/j.humov.2010.03.006 20650535

[B54] Piek J. P. Rigoli D. Pearsall-Jones J. G. Martin N. C. Hay D. A. Bennett K. S. (2007). Depressive symptomatology in child and adolescent twins with attention-deficit hyperactivity disorder and/or developmental coordination disorder. *Twin Res. Hum. Genet.* 10 587–596. 10.1375/twin.10.4.587 17708700

[B55] Prinstein M. J. Rancourt D. Adelman C. B. Ahlich E. Smith J. Guerry J. D. (2018). “Peer status and psychopathology,” in *Handbook of Peer Interactions, Relationships, and Groups*, 2nd Edn, eds Bukowski W. M. Laursen B. Rubin K. H. (New York, NY: The Guildford Press), 617–636.

[B56] Rigoli D. Kane R. T. Mancini V. Thornton A. Licari M. Hands B. (2017). The relationship between motor proficiency and mental health outcomes in young adults: a test of the environmental stress hypothesis. *Hum. Mov. Sci.* 53 16–23. 10.1016/j.humov.2016.09.004 27697306

[B57] Sanderson J. A. Siegal M. (1995). Loneliness and stable friendship in rejected and nonrejected preschoolers. *J. Appl. Dev. Psychol.* 16 555–567. 10.1016/0193-3973(95)90004-7

[B58] Schmidt M. E. Bagwell C. L. (2007). The protective role of friendships in overtly and relationally victimized boys and girls. *Merrill Palmer Q.* 53 439–460. 10.1353/mpq.2007.0021

[B59] Schneider B. H. (2016). *Childhood Friendships and Peer Relations: Friends and Enemies*, 2nd Edn Abingdon: Routledge.

[B60] Schoemaker M. M. Flapper B. Verheij N. P. Wilson B. N. Reinders-Messelink H. A. de Kloet A. (2006). Evaluation of the developmental coordination disorder questionnaire as a screening instrument. *Dev. Med. Child Neurol.* 48 668–673. 10.1017/S001216220600140X 16836779

[B61] Schoemaker M. M. Kalverboer A. F. (1994). Social and affective problems of children who are clumsy: how early do they begin? *Adapt. Phys. Activ. Q.* 11 130–140. 10.1123/apaq.11.2.130

[B62] Schoemaker M. M. Wilson B. N. (2015). “Screening for developmental coordination disorder in school-age children,” in *Developmental Coordination Disorder and its Consequences*, ed. Cairney J. (Toronto: University of Toronto Press), 169–191.

[B63] Skinner R. A. Piek J. P. (2001). Psychosocial implications of poor motor coordination in children and adolescents. *Hum. Mov. Sci.* 20 73–94. 10.1016/S0167-9457(01)00029-X 11471399

[B64] Smyth M. M. Anderson H. I. (2000). Coping with clumsiness in the school playground: social and physical play in children with coordination impairments. *Br. J. Dev. Psychol.* 18 389–413. 10.1348/026151000165760

[B65] Sullivan H. S. (1953). *The Interpersonal Theory of Psychiatry.* New York, NY: W W Norton & Co.

[B66] Tal Saban M. Kirby A. (2019). Empathy, social relationship and co-occurrence in young adults with DCD. *Hum. Mov. Sci.* 63 62–72. 10.1016/j.humov.2018.11.005 30503983

[B67] Taylor M. Hulette A. C. Dishion T. J. (2010). Longitudinal outcomes of young high-risk adolescents with imaginary companions. *Dev. Psychol.* 46 1632–1636. 10.1037/a0019815 20677857 PMC3353747

[B68] Traub A. (2006). Kontinuität und Kompensation. Die Bedeutung von Familie und Gleichaltrigen (Peers) für Persönlichkeit und Problemverhalten in der mittleren Kindheit. *Diskurs Kindheits Jugendforschung* 1 197–216.

[B69] Uchino B. N. (2009). Understanding the links between social support and physical health: a life-span perspective with emphasis on the separability of perceived and received support. *Perspect. Psychol. Sci.* 4 236–255. 10.1111/j.1745-6924.2009.01122.x 26158961

[B70] Wagner M. Bös K. Jascenoka J. Jekauc D. Petermann F. (2012). Peer problems mediate the relationship between developmental coordination disorder and behavioral problems in school-aged children. *Res. Dev. Disabil.* 33 2072–2079. 10.1016/j.ridd.2012.05.012 22750362

[B71] Wagner M. Jekauc D. Worth A. Woll A. (2016). Elaboration of the environmental stress hypothesis–results from a population-based 6-year follow-up. *Front. Psychol.* 7:1904. 10.3389/fpsyg.2016.01904 28018254 PMC5156825

[B72] Wilson B. N. Crawford S. G. Green D. Roberts G. Aylott A. Kaplan B. J. (2009). Psychometric properties of the revised developmental coordination disorder questionnaire. *Phys. Occup. Ther. Pediatr.* 29 182–202. 10.1080/01942630902784761 19401931

[B73] Wustmann Seiler C. Müller E. Simoni H. (2017). The protective role of childcare quality for behavioral adjustment in 3- to 5-year-old children. *Z. Entwicklungspsychol. Pädagog. Psychol.* 49, 1–10. 10.1026/0049-8637/a000162

[B74] Zwicker J. G. Suto M. Harris S. R. Vlasakova N. Missiuna C. (2018). Developmental coordination disorder is more than a motor problem: children describe the impact of daily struggles on their quality of life. *Br. J. Occup. Ther.* 81 65–73. 10.1177/0308022617735046

