# Corrigendum: Poor Motor Performance – Do Peers Matter? Examining the Role of Peer Relations in the Context of the Environmental Stress Hypothesis

**DOI:** 10.3389/fpsyg.2020.01206

**Published:** 2020-06-23

**Authors:** Olivia Gasser-Haas, Fabio Sticca, Corina Wustmann Seiler

**Affiliations:** ^1^Marie Meierhofer Children's Institute, Associated Institute of the University of Zurich, Zurich, Switzerland; ^2^Department of Pre-primary and Lower Primary Level, Zurich University of Teacher Education, Zurich, Switzerland

**Keywords:** motor performance in daily activities, internalizing problems, peer problems, popularity, friendship quality

In the original article, there was a mistake in Figure 3 as published. Two numbers in the figure, −0.44 and 0.04, are incorrect. The corrected Figure 3, with the corrected numbers −0.42 and 0.06, appears below.

**Figure 3:**
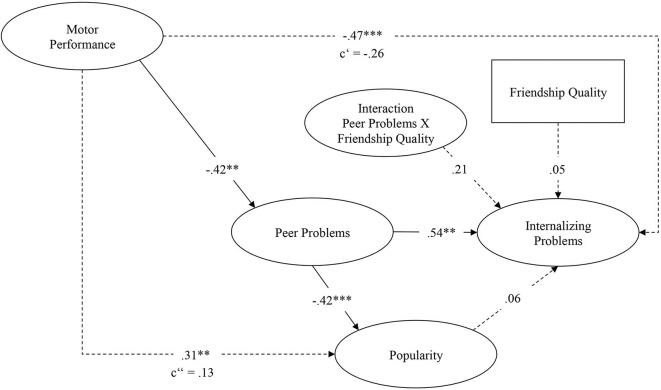
Standardized results of the structural equation model. Model fit (χ^2^ = 160.52; *df* = 147; CFI = 0.97; RMSEA = 0.02; SRMR = 0.05). *p < 0.05; **p < 0.01; ***p < 0.001.

The authors apologize for this error and state that this does not change the scientific conclusions of the article in any way. The original article has been updated.

